# Experimental Studies of the Attention Processing Model in Multiple Object Tracking Task

**DOI:** 10.3390/brainsci12121686

**Published:** 2022-12-08

**Authors:** Shuyi Liang, Yaning Guo, Sizhe Cheng, Shengjun Wu, Xiuchao Wang, Xinlu Wang, Diyan Lu, Xufeng Liu

**Affiliations:** Department of Military Medical Psychology, Air Force Medical University, Xi’an 710032, China

**Keywords:** attention, multi-focus theory, multiple object tracking (MOT) task, simultaneous–sequential paradigm

## Abstract

(1) Background: Attention is an important cognitive process in daily life. However, limited cognitive resources have been allocated to attention, especially for multiple objects and its mechanism is still unclear. Most of the previous studies have been based on the static attention paradigms with relatively lower ecological validity. Thus, we aimed to explore the attention processing mechanism in a multiple object tracking (MOT) task by using a dynamic attention paradigm. Two experiments were conducted to assess whether there was a multi-focus attention processing model, and whether the processing model changes with the number of target balls. (2) Methods: During the experiments, 33 university students completed MOT combined with the simultaneous–sequential paradigm, with tracking accuracy and reaction time of correct reaction as indicators. (3) Results: (i) When there were two target balls, an obvious bilateral field advantage was apparent. (ii) When there were four target balls, participants’ performance was significantly better when stimuli were presented simultaneously than when they were presented sequentially, showing a multi-focus attention processing model. (4) Conclusion: Attention processing is characterized by flexibility, providing strong evidence to support the multi-focus theory.

## 1. Introduction

With the continuous improvement in the level of social informatization, people’s intake of information is increasing, which occupies increasingly more cognitive resources. However, it has been agreed that our brain has limited capacity to process information at one time. Therefore, study on the mechanism of multi-object cognition is helpful to accurately select and process effective information. Attention plays an important role in the link between perception and cognition [1], and spatial attention has always been the focus in the field of attention [2,3,4], which refers to the process of allocating attention resources according to specific spatial information (e.g., location). Whether the brain can allocate attention to discrete spatial locations at the same time, or whether the focus of attention can be split, is one of the most important issues in the study of the attention processing mechanism. However, currently no consistent conclusion has been reached on this issue. Two key problems deserve attention. At first, the proper understanding of the processing mode of attention is critical for evaluating and training attention ability. Furthermore, attention mechanism has been widely used in various fields of artificial intelligence, such as object tracking, facial recognition, and further for self-driving cars and complex surveillance systems [5], which has been proven to be beneficial to improve the performance of the model. Bengio proposed that attention was a core element of “conscious” artificial intelligence in the report of ICLR 2020 [6]. Therefore, it is necessary to reveal the multi-object attention mechanism.

In 1980, Ponser put forward the “spotlight theory” (also referred to as attentional focus) [7], that creatively compared visual spatial attention to a spotlight, and suggests that an object in the spotlight is processed more effectively. It holds that attentional foci are distributed in a continuous spatial area and are inseparable [8]. Since then, the debate on whether the focus of attention could be split has begun. Several theories supported that the focus of attention was indivisible. For example, the model by Eriksen et al. (1974) also assumes that attention is distributed in a continuous spatial area [9]. Moreover, McCormick et al. (1998) found that most research has shown that the attention processing model still supports the “unified model” of attention (attention adjustment is limited to an inseparable continuous area) [10]. Further, several behavioral [9,11,12,13,14] electrophysiological [15,16,17] studies have supported the “unitary focus” attention processing model. In the past 15 years, however, more empirical studies have shown that the attention processing model can be parallel and “multi-focus”. For example, Awh et al. (2000) first found that study participants could allocate attention to 2–4 discontinuous positions with the method of partial report [18]. The experiment by Alvarez (2005) proved that when stimuli are simultaneously presented in the left and right visual hemifields, twice as many stimuli can be detected compared to when they are presented in a single visual hemifield. It was concluded that the attention process in the left and right visual hemifields are independent of each other. This effectively refuted the idea of a unitary focus, and put forward the multi-focus theory [19]. In a multi-object visual tracking task, a study participant can simultaneously track about four objects. Although this paradigm is different from the traditional attention allocation paradigm, some researchers believe that the ability to track multiple objects simultaneously supports the multi-focus attention processing model [20,21]. In addition, studies using event-related potentials and fMRI (functional magnetic resonance imaging) show that in tasks that require simultaneous attention allocation to multiple stimuli, the brain signals induced by the objects are enhanced, while the signals induced by a single distractor located between two objects are suppressed. This evidence shows the great flexibility of attention allocation [22,23], and opposes the unitary nature of attention focus. Although a growing number of studies supported the existence of multi-focus attention, researchers still believe that the multi-focus processing method focusing on multiple objects simultaneously consumes more attention resources and requires higher attention costs. Therefore, it cannot be the first choice in daily life. In the literature review by Jans et al. (2010), the following four criteria of focus splitting were put forward: (1) the task should be appropriately difficult; (2) the stimulus presentation time is short enough to prevent the generation of attention strategies (e.g., attention transfer); (3) there is appropriate clue-object interval: for simple tasks, short intervals should be adopted to prevent the early concentration and transfer of attention resources; for difficult tasks, long intervals should be adopted to ensure the thorough processing of clues; and (4) a complete assessment should be made of the attention of distractors surrounding the object to ensure they do not increase attention on the object. None of the 19 studies included in the review met all criteria [24]. Therefore, to date, there is no consistent conclusion regarding whether there is a multi-focus model during attention processing.

**Figure 1:**
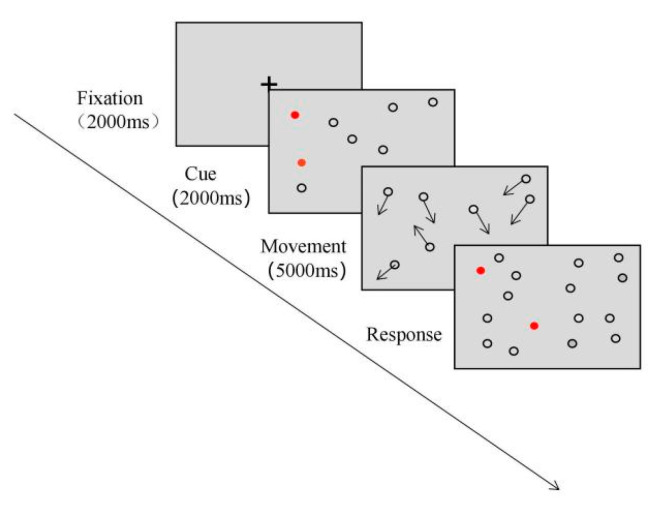
Classical MOT task (target number is four).

**Figure 2:**
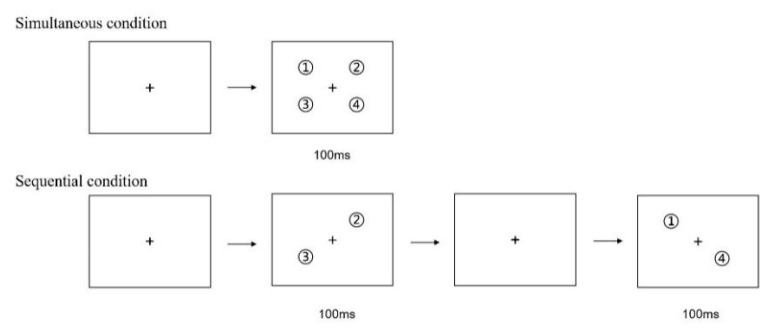
Simultaneous–sequential paradigm.

Since the 1980s, a large number of research based on the static attention paradigm has been accumulated in the field of spatial attention. However, attention exhibited a rhythmic and discrete temporal characteristic [25,26,27], with the temporal dimension involving dynamism. Therefore, it is necessary to reveal an attention processing mechanism with a dynamic paradigm. The multiple object tracking (MOT) task, proposed by Pylyshyn and Storm (1988) [21], is one of the classic paradigms for studying visual attention ability in dynamic scenes, which is often used to study the attention processing mechanism, attention allocation, and people’s differences in performance and training on MOT [28]. The classic MOT paradigm usually comprises three stages: (1) Cue: The cue stage involves the presentation of some simple objects with the same surface features (e.g., circles, squares, or the same letters). Some stimuli are marked as targets by flashing and changing some features, while others are distractors. (2) Movement: The cue disappears at this stage, all the objects start to move randomly and independently for several seconds, and observers are asked to track the marked targets during the cue stage; (3) Response: After several seconds of movement, movement stops and observers are asked to indicate which objects were targets (overall report) or whether a particular object was a target (partial report), and their response times and tracking accuracy rates are recorded (taking a target number of four as an example, as shown in Figure 1). Compared with traditional static attentional cognitive tasks, MOT has four characteristics of persistence, selectivity, dispersion, and dynamics [29], MOT is better aligned with the fact that people need to process a large quantity of information with limited attention resources, and it shows greater ecological validity [30]. Another paradigm used in this study is the simultaneous–sequential paradigm, which was first proposed by Eriksen and Spencer (1969) and used to study the processing speed of sensory–perceptual information [31]. Since then, many scholars have applied this paradigm to various studies regarding the resource constraints of visual perception and cognitive ability. For example, it was first used by Shiffrin and Gardner (1972) to study attentional ability [32]. In this paradigm, stimuli are presented in simultaneous and sequential time conditions: in the simultaneous condition, all stimuli are presented simultaneously while stimuli are presented successively in subsets in the sequential condition (as shown in Figure 2). Importantly, the presentation time of all stimuli is the same. The logic of this paradigm is that the processing load under simultaneous conditions is twice that under sequential conditions within the same processing time, if the performance of individual under simultaneous conditions is not worse than that under sequential conditions, it provides evidence for parallel, multi-focus processing.

**Figure 3:**
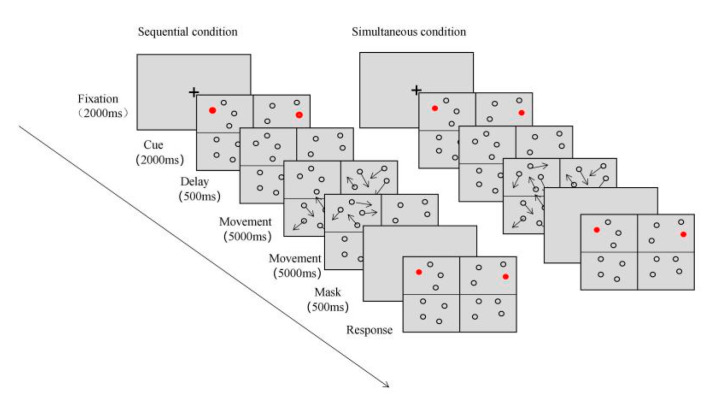
A Combination of MOT task and simultaneous–sequential paradigm.

To sum up, based on the literature review by Jans et al. (2010), there is still controversy about the existence of a multi-focus attention model, whether the foci of attention processing are split should be judged using sound criteria [24]. In addition, most of the previous studies on the attention processing model were based on the static attention cognitive paradigm, with relatively low ecological validity. Therefore, this study combined the MOT paradigm and the simultaneous–sequential paradigm and adapted the paradigms appropriately based on the criteria proposed by Jans et al. (2010) to study the existence of the multi-focus attention processing model with higher ecological validity (Figure 3) [24]. The multi-focus attention model predicts that attention processing is parallel, and stimuli are processed simultaneously and independently. By comparison, a unitary-focus attention model predicts that attention processing is serial, and stimuli are processed successively. In the simultaneous condition, the number of stimuli to be processed at any one time is twice that of the sequential condition. If the multi-focus attention model does not exist and study participants cannot choose two or more nonadjacent positions at the same time, the simultaneous condition will be more difficult than the sequential condition. Therefore, in this study, it was assumed that the MOT ability is at least no worse when stimuli are presented simultaneously than that when stimuli are presented sequentially, and multi-focus attention exists. This study aimed to explore: (1) when there are two objects, whether there is bilateral independence in more difficult and higher ecological validity tasks, which could provide evidence for multi-focus patterns of attention; (2) the attention processing model when there are two objects; and (3) whether the attention processing model changes with an increased number of objects.

## 2. Experiment I

### 2.1. Methods

#### 2.1.1. Participants

A total of 33 students (17 males and 16 females) with an average age of 23.32 ± 3.03 years from a university in Shaanxi were recruited as research participants. All participants had normal vision or corrected vision, normal color perception, and were right-handed. All participants volunteered to participate in the experiment, they signed an informed consent form before the experiment commenced, and received a reward with small cash after it was completed.

#### 2.1.2. Experiment Design

This study adopted a 2 (spatial conditions: between/within hemifields) ×2 (time conditions: simultaneous/sequential presentation) within-subjects design. The dependent variables were the accuracy and reaction times of participants. The tracking accuracy was the ratio of the number of objects correctly selected by participants to the total number of objects, and the total accuracy was the average of each trial. Only when the reaction was correct, the reaction time was included in the calculation. Both indicators were coded and calculated by MATLAB.

#### 2.1.3. Instruments and Materials

All experimental programs were written by Matlab2017a and psychtoolbox (version 3). The stimuli were presented on a Dell P1917s display, with a 19-inch screen, screen resolution of 1280 × 1024 Hz, and vertical refresh rate of 60 Hz, which ensures smooth and clear presentation of stimuli. The whole experiment was completed in a quiet, well-lit laboratory. The participant sat in front of the screen, and his/her chin was fixed by a chin rest to ensure that the central axis of the head was aligned with the center of the screen, and the vertical distance between the eyes and the screen was 70 cm.

In the center of the screen, a square area separated by a black frame was presented as the tracking area both with a length and width of 35.2° visual angle. The stimulus materials used in the experiment included (1) a cross gaze point composed of vertical and horizontal line segments with a length of 2° visual angle; (2) balls with the same shape and size and a radius of 0.53° visual angle. Programming with the collision detection algorithm ensured the balls would not be blocked during the motor process, and would randomly change the motor direction after collision. The distance between the ball and the frame would not be less than a ball’s diameter.

#### 2.1.4. Experimental Process

At the beginning of the experiment, instructions were presented on the screen, the subjects were asked to track the target balls that had been marked by turning red, and there was no further suggestive information. Then, an 80 trial adaptive stage was written by the QUEST program in psychtoolbox [33,34]. After each participant completed the adaptive task, a distribution chart was obtained, with the ordinate denoting the accuracy and the abscissa denoting the number of trials, which used a maximum likelihood method and a Bayesian algorithm to calculate the velocity of the ball when each subject achieved an accuracy of 75%. This stage took about 20 min to complete. Each participant participated in the formal experiment at the speed of the adaptive test, which consisted of 120 trials and took about 25 min. To avoid the generation of fatigue, the adaptive and formal experiments were completed in two separate sessions, and the interval was two weeks. In addition, an unlimited rest period occurred every 20 trials, and when the subjects finished their rest, they could press the button to continue the experiment.

**Figure 4:**
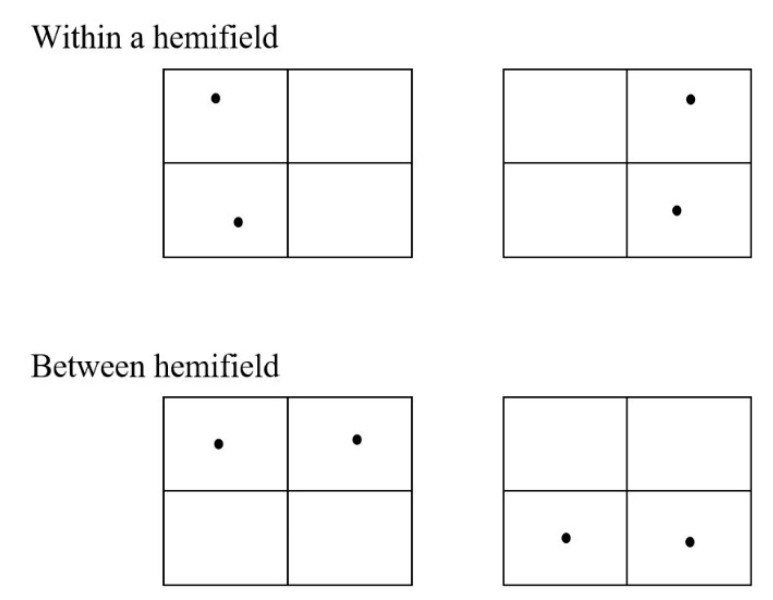
Examples of four presentations of the target ball.

The experimental paradigm was the combination of the MOT task and the simultaneous–sequential paradigm. The time condition was controlled by the sequence of stimulus presentation. In the simultaneous condition, the balls in four quadrants moved simultaneously, while the diagonal quadrants (the first and third quadrants, the second and fourth quadrants) moved in sequence in the sequential condition. The spatial condition was controlled by the quadrant of stimulus presentation, involving four situations as shown in Figure 4:

#### 2.1.5. Statistical Analysis

First, the data were screened to exclude data with reaction times exceedingly more than 3 standard deviations from the mean so that the data for all participants conformed to a normal distribution. Second, the behavioral data were analyzed using the statistical software SPSS 22.0, mainly using the repeated measurement ANOVA.

### 2.2. Results

#### 2.2.1. Tracking Accuracy

**Figure 5:**
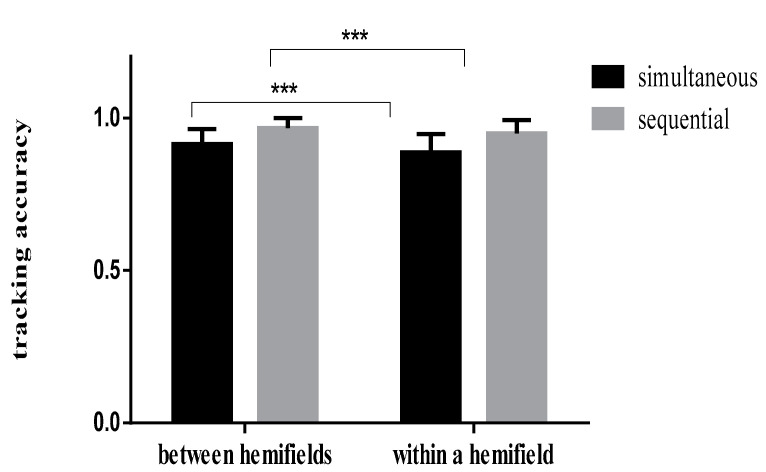
Comparison of tracing accuracy of different presentation conditions under simultaneous and sequential conditions. Note: *** indicates the significance level is *p* < 0.001.

Tracking accuracy rates in the different conditions are shown in Table 1. Results of the repeated measurement analysis of variance (ANOVA) of two (spatial conditions: between visual hemifields/within a visual hemifield) × 2 (time conditions: simultaneous/sequential presentation) showed that the main effect of time was significant (F (1,30) = 11.929, *p* = 0.002, η_p_^2^ = 0.284), and participants’ accuracy was higher in the sequential condition than in the simultaneous condition. Similarly, the main effect of the spatial condition was significant (F (1,30) = 60.377, *p* < 0.001, η_p_^2^ = 0.668), and participants’ accuracy was higher when stimuli were presented between visual hemifields than that when stimuli were presented within a visual hemifield. The interaction between time and space was not significant (Figure 5).

#### 2.2.2. Tracking Reaction Time

**Figure 6:**
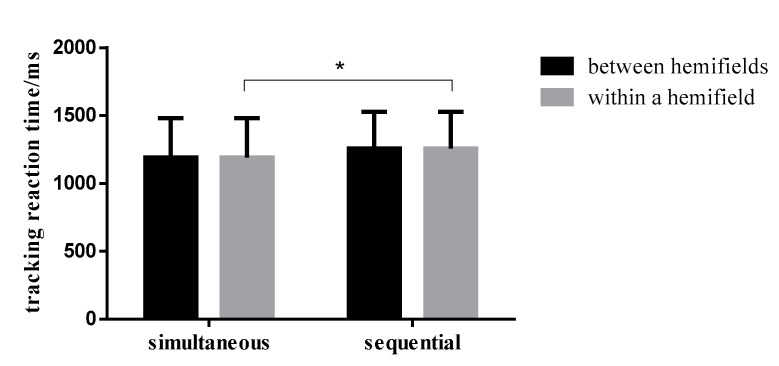
Comparison of tracing reaction time of different presentation conditions under simultaneous and sequential conditions. Note: * indicates the significance level is *p* < 0.05.

The tracking reaction times in the different conditions are shown in Table 2. Results of the repeated measurement ANOVA of 2 (spatial conditions: between visual hemifields/within a visual hemifield) × 2 (time conditions: simultaneous/sequential presentation) showed that the main effect of time was not significant for reaction times while the main effect of the spatial condition was significant (F (1,30) = 5.714, *p* = 0.023, η_p_^2^ = 0.160), and participants’ reaction times were higher when stimuli were presented between visual hemifields than when they were presented within a visual hemifield. It can be seen that the interactions between the time and spatial conditions were also significant (F (1,30) = 5.196, *p* = 0.030, η_p_^2^ = 0.148). Further simple effects analysis showed that when stimuli were presented between visual hemifields, the reaction times were lower when stimuli were presented simultaneously than when presented sequentially, and the difference was not statistically significant. By comparison, when stimuli were presented within a visual hemifield, the reaction times were higher when stimuli were presented simultaneously than when presented sequentially, and the difference was statistically significant (F (1,30) = 4.783, *p* = 0.037, η_p_^2^ = 0.138) (Figure 6).

### 2.3. Summary

The results showed that, when the time condition was a simultaneous presentation, the accuracy of stimulus reactions presented between visual hemifields was significantly higher than when presented within a unitary visual hemifield, with a shorter reaction time, showing a clear bilateral field advantage [35]. In other words, when the information was distributed in the left and right visual hemifields, the processing performance was better than when it was presented in a unitary visual hemifield. However, when the time condition was the sequential presentation, there was no obvious bilateral advantage and the accuracy of stimulus reactions presented between visual hemifields was higher than when it was presented within a unitary visual hemifield, but the reaction time was longer. In this case, high accuracy was probably caused by slower reaction times. The possible reason is that in the sequential presentation, the participant only needs to process one object in each motor process, and the maximum threshold of attention resources is not reached, and high load is the premise of bilateral field advantage [35]. In Experiment I, the reaction performance in the simultaneous condition was significantly worse than in the sequential condition, and there was no evidence that directly supports the existence of the multi-focus attention model, which is inconsistent with previous research results [36]. One possible reason is that the attention resources were not completely occupied during the simultaneous or sequential tracking of two objects, which does not meet the premise of focus splitting—the concentration of attention resources [24]. Therefore, in Experiment II, the number of balls was increased to 4 and 6, to observe whether the multi-focus attention model would occur.

## 3. Experiment II

### 3.1. Methods

#### 3.1.1. Participants

The same as Experiment I.

#### 3.1.2. Experiment Design

This study adopted a 2 (time conditions: simultaneous/sequential presentation) × 3 (number of target balls: 2, 4, 6) within-subjects design. The dependent variables were the answer accuracy and reaction times of the participants. The tracking accuracy was the ratio of the number of targets correctly selected by participants to the total number of targets, and the total accuracy was the average of each trial.

#### 3.1.3. Instruments and Materials

The same as Experiment I.

#### 3.1.4. Experimental Process

As in Experiment I, the experiment consisted of an adaptive test (80 trials) and a formal test (120 trials), with a total duration of about 50 min.

#### 3.1.5. Statistical Analysis

The same as Experiment I.

### 3.2. Results

#### 3.2.1. Tracking Accuracy

**Figure 7:**
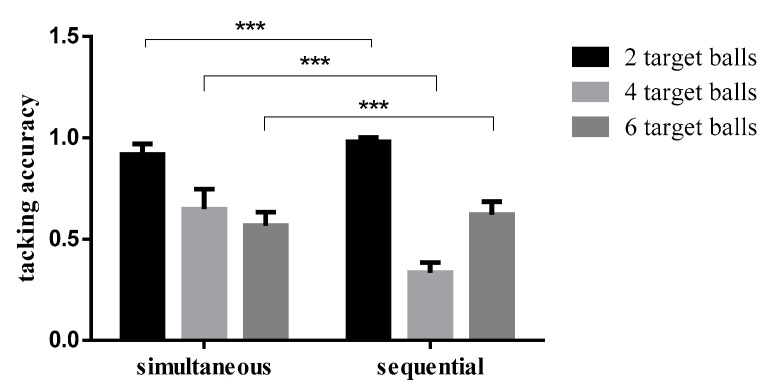
Comparison of the accuracy rate of different numbers of target balls in the simultaneous and sequential conditions. Note: *** indicates the significance level is *p* < 0.001.

Tracking accuracy rates in different conditions are shown in Table 3. According to the repeated measurement variance analysis of 2 (time condition: simultaneous/sequential presentation) × 3 (number of target balls: 2/4/6) on the tracking accuracy, the main effect of time condition was significant (F (1,32) = 81.410, *p* < 0.001, η_p_^2^ = 0.718; the main effect of the number of balls was significant (F (2,32) = 1125.138, *p* < 0.001, η_p_^2^ = 0.972); the interaction between time condition and the number of balls was significant (F (2,64) = 347.826, *p* < 0.001, η_p_^2^ = 0.916). The results of the simple effect test showed that when there were two targets, the tracking accuracy in the sequential condition was higher than that in the simultaneous condition, and the difference was statistically significant (F (1,32) = 61.371, *p* < 0.001, η_p_^2^ = 0.657). When there were four targets, the tracking accuracy in the simultaneous condition was higher than that in the target condition, and the difference was statistically significant (F (1,32) = 445.060, *p* < 0.001, η_p_^2^ = 0.933). When there were six targets, the tracking accuracy in the sequential condition was higher than that in the simultaneous condition, and the difference was statistically significant (F (1,32) = 19.765, *p* < 0.001, η_p_^2^ = 0.382) (Figure 7).

#### 3.2.2. Tracking Reaction Time

**Figure 8:**
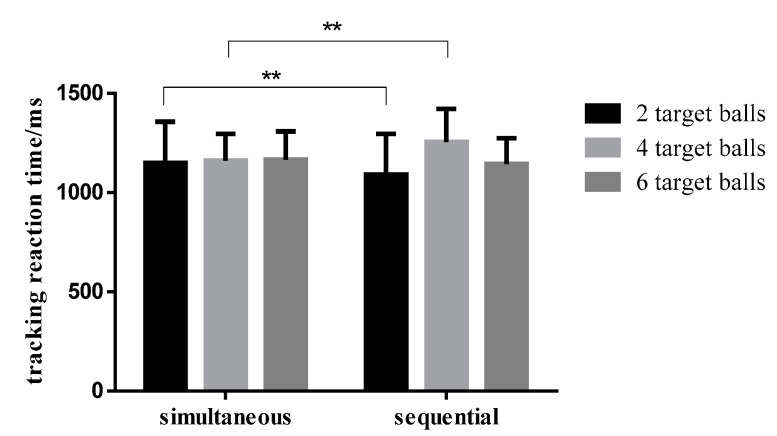
Comparison of the accuracy rate of different numbers of target balls in simultaneous and sequential conditions. Note: ** indicates the significance level is *p* < 0.01.

The tracking reaction time in different conditions is shown in Table 4. According to the repeated measurement variance analysis of 2 (time condition: simultaneous/sequential presentation) × 6 (number of target balls: 2/4/6) on the reaction time, the main effect of time condition was not significant; the main effect of the number of balls was significant (F (1,32) = 11.690, *p* < 0.001, η_p_^2^ = 0.430); the interaction between time condition and the number of balls was significant (F (1,32) = 9.461, *p* = 0.001, η_p_^2^ = 0.379). Further simple effect analysis on the interaction shows that, when there were two targets, the reaction time in the sequential condition was lower than that in the simultaneous condition, and the difference was statistically significant (F (1,32) = 22.797, *p* < 0.001, η_p_^2^ = 0.416). When there were four targets, the reaction time in the simultaneous condition was lower than that in the target condition, and the difference was statistically significant (F (1,32) = 8.682, *p* = 0.006, η_p_^2^ = 0.213). When there were six targets, the reaction time in the sequential condition was lower than that in the simultaneous condition, although it was not statistically significant (Figure 8).

### 3.3. Summary

The results showed that when there were two target balls, the tracking accuracy in the sequential condition was significantly higher than in the simultaneous condition, with shorter reaction times, which was consistent with the results in Experiment I. Thus, there was no evidence that directly supported the multi-focus attention model. When there were four targets, however, the tracking performance in the simultaneous condition was significantly better than in the sequential condition, which supported the existence of multi-focus attention. When the number of targets increased to six, the performance in the sequential condition was significantly better than in the simultaneous condition. With the increase in target balls, the attention model in the MOT task changed. In other words, the multi-focus attention model was only evident in this study when there were four targets.

## 4. Discussion

This study combined the MOT task and the simultaneous–sequential paradigm to study the attention processing model, and to explore whether there is a parallel and multi-focus processing model of stimuli when there are two objects, under the MOT task and in two stimulus presentation time conditions (Experiment I). An additional question was whether this processing model changed with more objects (Experiment II). The unitary-focus model predicts that in MOT tasks, only one object can be coded at a time, and by quickly switching attention foci among multiple objects [8], multiple objects are coded and processed. The multi-focus model predicts that individuals can process multiple objects both simultaneously and independently. Therefore, the logic of this paradigm is that the load of stimuli presented simultaneously in the two time conditions is twice that of stimuli presented sequentially, which makes it more difficult to track objects [32]. In view of this, the unitary-focus model predicts that stimuli presented simultaneously results in poorer performance, while the multi-focus model predicts that a person’s performance when stimuli are presented simultaneously is at least no worse than when stimuli are presented sequentially.

In Experiment I, the main effect of the spatial condition was significant, and the existence of bilateral field advantage was observed, which is consistent with previous research results [19,36]. There are independent attention resources in the two visual hemifields for visual tracking, and the attention process plays a key role in the emergence of a bilateral field advantage [37], which provides some evidence for the split of attention foci. The main effect of the time condition was also significant, and the performance in the sequential condition was significantly better than in the simultaneous condition. There was no evidence that directly supports the existence of a multi-focus attention processing model, which was contrary to the experimental expectation. The possible reason for this result is that during the MOT process, participants could track 4–5 balls simultaneously, with an accuracy rate of up to 85–95% [19]. In this part of experiment, there were only two objects, and the average accuracy reached 92.8%. The attention resources were not completely occupied, which does not meet the criteria for focus splitting—the concentration of attention resources [21]. Therefore, in Experiment II, the number of balls was increased to 4 and 6, to observe whether the attention processing model changed when resources were completely occupied and overloaded.

In Experiment II, the interaction between the time condition and the number of target balls was significant. When there were two target balls, the performance in the sequential condition was significantly better than in the simultaneous condition, which was inconsistent with previous research results [36]. One possible reason for this result is that the stimuli used in the study by Howe et al. (2010) were composed of lattices of four dots, and the mode of motion was one-dimensional clockwise or counterclockwise. In Experiment II, however, the motion of balls was two-dimensional, and the direction and speed of motion were random and unpredictable. When there were four target balls, the performance in the simultaneous condition was significantly better than in the sequential condition, which is consistent with previous research results and provides evidence that supports the multi-focus attention model [36]. When there were six target balls, the performance in the sequential condition was significantly better than in the simultaneous condition, and the attention processing model was changed into the unitary-focus and serial model. A possible reason for this result is that the number of balls exceeded the maximum attention threshold. In this case, individuals actively adopt strategies to track the targets to reach the highest efficiency, such as tracking adjacent target balls and abandoning the distant target balls. Results of Experiment II show that the attention processing model is not unitary and constant, but somewhat flexible.

It is obvious that working memory and visual spatial attention are strongly correlated [38]. In the present study, the working memory load of the subjects may be higher under the sequential condition than that under the simultaneous condition. According to the resource limitation theory, when two tasks need to occupy the same resources, the performance will decline [39], which may lead to another interpretation of the experimental results. However, previous study has shown that spatial and non-spatial working memory are dissociable functions of the brain [40]. Howard et al.’s study (2020) used MOT task, and found that for a purely spatial task, working memory and visual spatial attention appear to recruit separate capacity-limited resources [41]. Besides, several studies have also suggested that the resources of spatial working memory and visual spatial attention are independent of each other [42,43,44,45]. Further, Olivers proposed a model, which suggested that when the target is new on every trial, the effect of memory items is weak or absent [46], which is consistent with the situation in this study. Thus, we partially excluded the influence of working memory. Nevertheless, there is no denying of the strong relationship between working memory and attention. Therefore, the results may be confounded by the different components of working memory, and future research could further isolate the effect of this factor, such us combining event-related potential (ERP) and other technologies.

The current research has important theoretical implications for our understanding of visual processing, which can only be achieved by correctly understanding the processing mode of attention, and is the basis for the selection and training of attention ability of personnel with special occupational needs and further mechanism exploration. Moreover, this finding may be applied to the optimization of artificial intelligence models, such as object tracking.

## 5. Limitations

Despite the novelty of the current findings that indicate the attention processing mechanism is flexible, many strategies (such as switching and biased selection) can be adopted in the attention process. This study was based on behavioral data only, however, eye tracking and ERP technology can be added in future research to study the attention processing model more comprehensively and rule out the influence of working memory. Furthermore, we only examined the regular processing pattern of attention, but whether the processing helps track accuracy should be further investigated in future research.

## 6. Conclusions

In sum, evidence for the existence of a multi-focus and parallel attention processing model was found in the studies reported in this paper. When there were two target balls, there was an obvious bilateral field advantage. However, when the number of target balls just reached the threshold of four, the performance of study participants was clearly better when stimuli were presented simultaneously than when they were presented sequentially, providing strong evidence that supports the multi-focus theory.

## Figures and Tables

**Table 1:** Tracking accuracy (M ± SD, N = 33).

	Spatial Conditions	Between Hemifields	Within Hemifields
Time Conditions			
Simultaneous presentation		0.914 ± 0.050	0.887 ± 0.060
Sequential presentation		0.967 ± 0.033	0.949 ± 0.044

**Table 2:** Tracking reaction time t/ms (M ± SD, N = 33).

	Spatial Conditions	Between Hemifields	Within Hemifields
Time Conditions			
Simultaneous presentation		1190.054 ± 292.647	1259.275 ± 270.441
Sequential presentation		1207.443 ± 305.609	1205.374 ± 332.441

**Table 3:** Tracking accuracy (M ± SD, N = 33).

	Spatial Conditions	2	4	6
Time Conditions				
Simultaneous presentation		0.918 ± 0.053	0.648 ± 0.099	0.567 ± 0.066
Sequential presentation		0.980 ± 0.022	0.334 ± 0.050	0.620 ± 0.065

**Table 4:** Tracking reaction time t/ms (M ± SD, N = 33).

	Spatial Conditions	2	4	6
Time Conditions				
Simultaneous presentation		1149.041 ± 208.266	1161.923 ± 133.103	1165.379 ± 142.492
Sequential presentation		1090.333 ± 205.785	1256.240 ± 165.229	1144.205 ± 129.677

## Data Availability

The datasets presented in this article are not readily available be- cause the datasets involve unfinished research projects. If necessary, requests to access the datasets should be made to the corresponding author.
